# Crocin Ameliorates Cognitive Impairment and Pathological Changes in Alzheimer's Disease Model Mice by Regulating Gut Microbiota

**DOI:** 10.1002/fsn3.71117

**Published:** 2025-10-21

**Authors:** Zhiyan Zou, Dan Lei, Xilian Wang, Yuan Yin, Hong Li, Xin Di, Xiaoan Li

**Affiliations:** ^1^ School of Life Science and Engineering Southwest University of Science and Technology Mianyang China; ^2^ NHC Key Laboratory of Nuclear Technology Medical Transformation, Mianyang Central Hospital, School of Medicine, University of Electronic Science and Technology of China Mianyang China; ^3^ School of National Defense Science and Technology College Southwest University of Science and Technology Mianyang China

**Keywords:** Alzheimers disease, crocin, gut microbiota, neuroinflammation

## Abstract

Alzheimer's disease (AD), a primary cause of dementia, places a significant strain on both patients and society due to the absence of effective treatments. Recent research suggests that the gut microbiota may play a role in the development of AD. Crocin, a compound derived from traditional medicine, has demonstrated potential in alleviating neurological disorders and influencing gut microbiota, yet its specific mechanisms in AD remain unclear. In this study, we administered Crocin or saline to 5xFAD mice and wild‐type controls. We discovered that Crocin treatment led to notable improvements in cognitive function, as measured by the Morris water maze test, reduced beta‐amyloid (Aβ) accumulation, and decreased neuroinflammation, as indicated by reduced microglial and astrocyte activation. Metagenomic sequencing revealed a significant increase in the gut microbiota diversity, specifically the abundance of *Firmicutes*, *Verrucomicrobiota*, and *Akkermansia*. Additionally, Crocin enhanced intestinal barrier function by upregulating tight junction proteins and Secretory immunoglobulin A, while improving the structure of the jejunal mucosa. These results suggest that Crocin may alleviate cognitive deficits and neuropathological changes in 5xFAD mice, possibly through modulation of the gut microbiota and strengthening the gut barrier, presenting it as a promising therapeutic approach for AD.

## Graphical abstract

This research provides novel insights into the role of the gut‐brain axis in the pathogenesis of AD and highlights Crocin's potential as a therapeutic candidate for improving both cognitive function and pathological outcomes in AD. We believe these findings have significant implications for understanding the interplay between gut microbiota and neuroinflammation in the context of neurodegenerative diseases and may pave the way for novel therapeutic strategies.

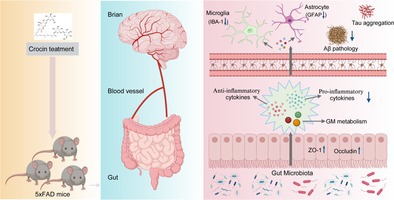

## 1 Introduction

Alzheimer's disease (AD) is a progressive neurodegenerative disorder characterized primarily by memory loss and cognitive decline, and it is most prevalent among elderly populations. The associated healthcare costs are substantial, severely impairing patients' quality of life and imposing a significant economic burden on families and society at large (Zhang et al. 2020). The pathogenesis of AD is multifactorial, involving several well‐established mechanisms. These include the deposition of beta‐amyloid (Aβ) plaques, the hyperphosphorylation of tau protein and formation of neurofibrillary tangles, neuroinflammation, cholinergic system dysfunction, and gut microbiota dysbiosis (Chen 2018; Liu, Gao, et al. 2020). Current therapeutic strategies primarily focus on Aβ clearance or provide symptomatic relief, with commonly used agents including aducanumab, lecanemab, and sodium oligomannate. However, these treatments are often costly, can induce significant side effects, are restricted to specific patient populations, and suffer from poor accessibility (Sevigny et al. 2016; van Dyck et al. 2023; Wang, Cai, et al. 2019). Consequently, the continued research and development of novel therapeutic approaches for the prevention, management, and treatment of AD remain urgently needed.

Traditional Chinese medicine (TCM) constitutes a comprehensive repository of therapeutic knowledge, primarily utilizing compound formulas, single herbs, and active ingredients to achieve therapeutic effects. Research has demonstrated that various TCM interventions can alleviate the pathological features of AD and other neurodegenerative disorders (Pei et al. 2020). These beneficial effects are achieved through multiple mechanisms, including the reduction of neuronal loss, enhancement of synaptic integrity, and attenuation of neuroinflammation via anti‐inflammatory and antioxidant properties. Additionally, TCMs can modulate key signaling pathways, such as the NF‐κB, JAK/STAT, and AMPK/mTOR signaling pathways (Ding et al. 2022). Therefore, the exploration of drugs or active ingredients derived from TCM represents a promising therapeutic strategy for AD. Crocin, a natural compound extracted from saffron ( *Crocus sativus* ), is a polar carotenoid that has been used in TCM (Maqbool et al. 2022). Research has demonstrated that Crocin exerts therapeutic effects against neurodegenerative diseases, including AD (Abdian et al. 2024). In an AD rat model induced by D‐galactose and aluminum chloride, Crocin was found to suppress oxidative stress by reducing superoxide dismutase activity and lowering glutathione peroxidase levels. Furthermore, it mitigated neuronal apoptosis and ameliorated learning and memory deficits (Wang, Sun, et al. 2019). In a study on 5xFAD mice, dietary administration of Crocin for 1 month significantly reduced the total Aβ levels in 5xFAD brains' hippocampi and decreased Aβ brain deposits (Batarseh et al. 2017). Current research on the therapeutic effects of Crocin in AD has predominantly concentrated on protein signaling pathways, yet there are no reports investigating its impact on the gut microbiota within AD models.

A growing body of evidence underscores the critical role of gut microbiota in the initiation and progression of AD (Jiang et al. 2017). A stable gut microbial ecosystem and intestinal integrity promote gut immune function, whereas dysbiosis of the gut microbiota may stimulate the production of endotoxins and other neurotoxic metabolites. These changes can disrupt the intestinal mucosal barrier and increase its permeability (Ogunrinola et al. 2020). These endotoxins also bind to specific receptors on immune cells, which stimulate the expression of pro‐inflammatory cytokines, including interleukin‐6 (IL‐6) and tumor necrosis factor‐α (TNF‐α), and triggers a systemic inflammatory response (Brown and Heneka 2024). This systemic inflammation can, in turn, induce neuroinflammation, thereby promoting the pathogenesis of neurodegenerative diseases. Furthermore, the gut microbiota directly and indirectly produces a range of metabolites, including short‐chain fatty acids (SCFAs) and lipopolysaccharides (LPS), as well as neurotransmitters, such as 5‐hydroxytryptamine (5‐HT) and gamma‐aminobutyric acid (GABA). These molecules can significantly influence the onset and progression of AD (Gomaa 2020; Zou et al. 2024).

Recent studies have indicated that TCM can ameliorate neurological diseases through the modulation of gut microbiota. Following oral administration, only a minor fraction of its small‐molecule compounds is directly absorbed into the systemic circulation; the majority of components proceed into the digestive tract, where they interact with the gut microbiota. These interactions subsequently alter the microbial community structure and metabolic output, ultimately mediating the therapeutic effects. Cui et al. demonstrated that Ganmaidazao decoction (GMDZ) improved the learning ability of AD rats, reduced Aβ protein deposition, decreased the abundance of pro‐inflammatory bacteria (e.g., *Shigella* and *Escherichia*), and restored gut microbiota diversity. Furthermore, metabolomic analysis revealed that GMDZ restored phenylalanine hydroxylase activity, reduced the abnormal accumulation of phenylalanine and phenylpyruvate, and consequently alleviated cognitive impairment in AD rats (Cui et al. 2023). In recent years, there has been an increasing emphasis on investigating the active components of TCM for the treatment of AD. For instance, Schisandra chinensis polysaccharide and oligosaccharide derived from Morinda officinalis have demonstrated potential in reversing gut microbiota dysbiosis, reducing brain inflammation, regulating endogenous metabolites, and providing therapeutic effects against AD (Fu et al. 2023; Chen et al. 2017). Fasina et al. discovered that Gastrodin enhances the memory of AD mice by increasing the abundance of *Lactobacillus* and *Firmicutes* bacteria, both of which are involved in the production of neurotransmitters like GABA, acetylcholine, and histamine, contributing to cognitive function (Fasina et al. 2022). A previous study observed that Curcumin administration significantly improved spatial learning and memory performance while also reducing the amyloid plaque burden in the hippocampus of APP/PS1 mice. Curcumin administration significantly altered the relative abundances of bacterial taxa, including *Bacteroidaceae*, *Prevotellaceae*, *Lactobacillaceae*, and *Rikenellaceae* at the family level, and *Prevotella*, *Bacteroides*, and *Parabacteroides* at the genus level, several of which have been identified as key bacterial species associated with AD development (Sun et al. 2020). In a study, depressive behavior in mice was found to improve after 6 weeks of treatment with Crocin, and 16S rRNA sequencing revealed that Crocin could modulate the composition of the gut microbiota in these mice (Xiao et al. 2019). Additionally, in a dextran sulfate sodium‐induced colitis mouse model, it was found that oral administration of Crocin inhibited the secretion of TNF‐α and IL‐6, regulated the gut microbiota composition, prevented the depletion of SCFAs, and improved intestinal barrier function (Banskota et al. 2021).

Based on established associations between Crocin, gut microbiota, and AD, we orally administered different doses of Crocin to 5xFAD mice to investigate whether it could ameliorate cognitive deficits by modulating the gut microbiota. Additionally, the secondary objective was to identify the optimal therapeutic dose of Crocin for AD.

## 2 Materials and Methods

### 2.1 Drugs and Reagents

Crocin (C_44_H_64_O_24_, purity > 98%) was obtained from Vicky Biotechnology Co. Ltd. (Chengdu, Sichuan, China). A 10 mg/mL solution of Crocin was prepared using physiological saline. Physiological saline (NaCl, 74%) and paraformaldehyde (PFA, 4%) were purchased from Chengdu Ruiya. The primary antibodies used for immunofluorescence and immunohistochemistry included Aβ1‐42 (Servicebio, HA721789, Wuhan, China), GFAP (Servicebio, GB11096‐50, Wuhan, China) IBA‐1 (Servicebio, GB15105‐50, Wuhan, China), zona occluden‐1 (ZO‐1) (Servicebio, GB111402‐100, Wuhan, China) and Occludin (Servicebio, GB111401‐100, Wuhan, China). The secondary antibodies included HRP‐conjugated goat anti‐rabbit IgG, CY3‐conjugated goat anti‐rabbit IgG, and Alexa Fluor 488‐conjugated goat anti‐mouse IgG, all of which were acquired from Servicebio Biotechnology Co. Ltd. The enzyme‐linked immunosorbent assay (ELISA) kits employed in this study were procured from Bioswamp Biotechnology Co. Ltd. and KANGKAIXIN Biotechnology Co. Ltd. These kits included the IL‐6, TNF‐α, mouse Secretory immunoglobulin A (sIgA), occludin, ZO‐1, and claudin‐4 ELISA Kit.

### 2.2 Animals and Grouping

**FIGURE 1:**
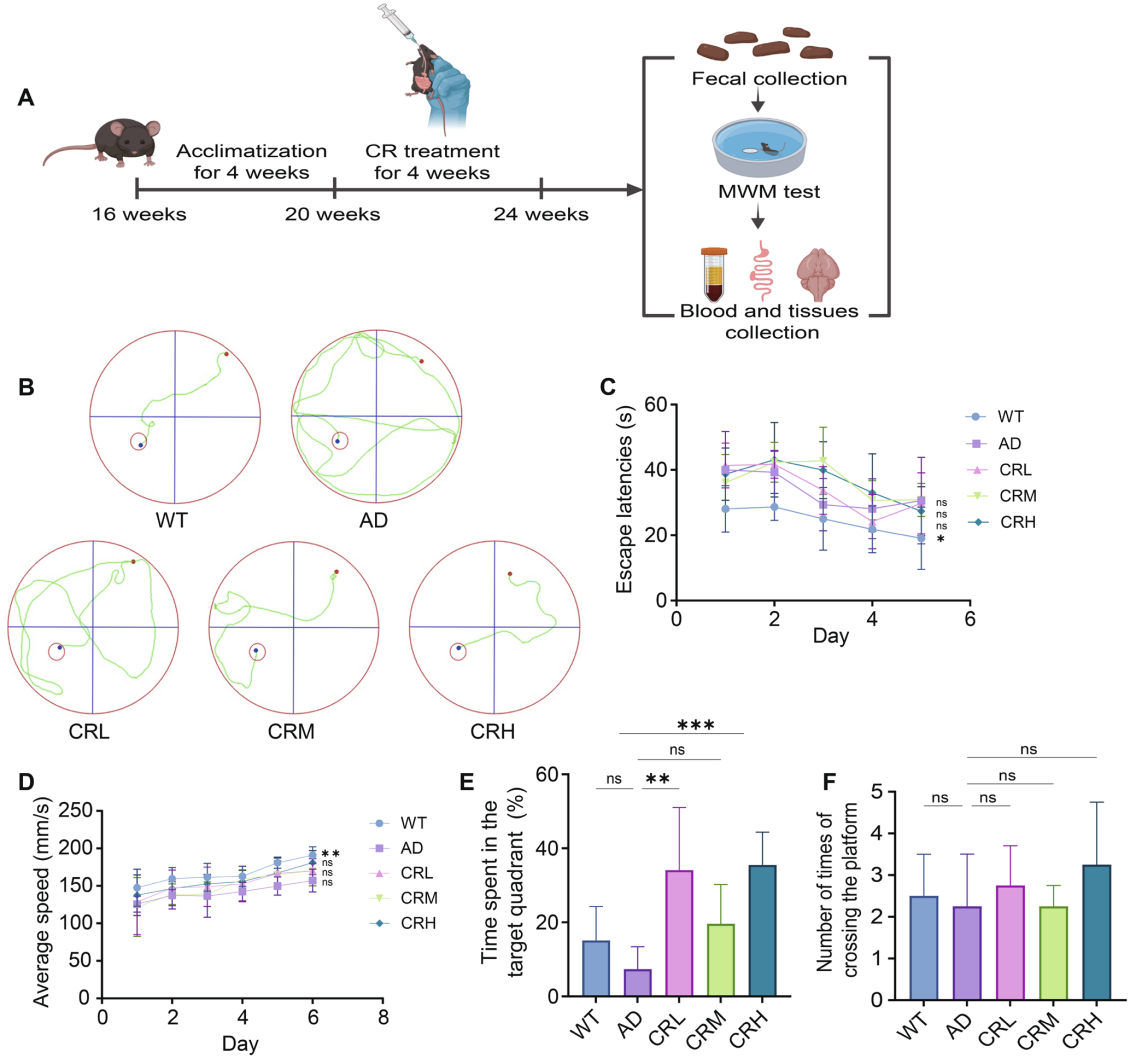
Crocin treatment ameliorated cognitive impairment in 5xFAD mice. (A) Experimental design. (B) Representative swimming trajectories during the test period. (C) The escape latency within 60 s during the test period. (D) The swimming average speed during the test period. (E) Percentage of time spent in the target quadrant during the test. (F) Platform crossing times within 60 s during the test period. Data are represented as the mean ± SD. Data were analyzed using ANOVA and followed by Tukey's multiple comparisons test. *p < 0.05, **p < 0.01, ***p < 0.001, ****p < 0.0001; ns, no statistical difference.

A total of 48 male 5xFAD mice (aged 4 months) and 12 wild‐type littermates were sourced from Cavens Laboratory Animal Co. Ltd. (Changzhou, Jiangsu, China) and housed in a specific pathogen‐free facility at Mianyang Central Hospital. The temperature was maintained at 25°C ± 2°C, with relative humidity controlled at 50% ± 5%, and a 12‐h light/dark cycle was followed for 4 weeks. Then, the male 5xFAD mice were randomly assigned to four experimental groups with 12 mice in each group: the Model group (AD, treated with 10 mg/kg physiological saline), the Crocin low dose group (CRL, treated with 10 mg/kg Crocin solution), the Crocin medium dose group (CRM, treated with 20 mg/kg Crocin solution), and the Crocin high dose group (CRH, treated with 40 mg/kg Crocin solution). Additionally, 12 wild‐type littermates were included as the control group (WT, treated with 10 mg/kg physiological saline). All treatments were administered via oral gavage once daily for 4 weeks. As shown in Figure 1A, after the treatment period, fecal samples were collected, behavioral tests were conducted, and the mice were anesthetized with 2.5% isoflurane in 100% oxygen at a flow rate of 1.5 L/min to obtain blood and tissue samples for further analysis. All animal experiments were approved by the Animal Ethics Committee of Mianyang Central Hospital (permission number: S20240303‐01). Simultaneously, all animal procedures were carried out following the Animal Research: Reporting of In Vivo Experiments (ARRIVE) guidelines.

### 2.3 Morris Water Maze Test

After 4 weeks of treatment, the spatial learning and memory functions were assessed using the Morris water maze (MWM) test. The detailed operational procedure for the MWM test has been previously described (Qian et al. 2022). The day preceding the experiment, the mice underwent a 24‐h acclimatization period in the water maze room. Over the course of five consecutive days of training, each mouse made four attempts per day to locate hidden underwater platforms in four distinct quadrants, each attempt lasting 60 s. On the sixth day of the experiment, the platform was removed. The mouse was placed in the water on the opposite side of the original platform and explored the platform's previous location. The water maze software recorded the number of crossings over the platform location, the time spent in the platform quadrant, the total distance traveled, the average speed, and the swimming trajectory during the training and testing phases, all of which were subsequently analyzed for further evaluation.

### 2.4 Metagenomic Sequencing

After gavage treatment, fresh fecal samples from all mice were collected and stored at −80°C. Metagenomic sequencing of the fecal samples from each group was performed by Beijing Novogene Bioinformatics Technology Co. Ltd. (Beijing, China). For sequencing, 1 μg of genomic DNA from each sample was randomly fragmented into ~350 bp fragments using a Covaris ultrasonicator, after which libraries were constructed. The library preparation process involved end repair, addition of A‐tailing, ligation of sequencing adapters, purification, and PCR amplification. Following library construction, preliminary quantification was carried out using a Qubit 2.0 fluorometer. The library was diluted to 2 ng/μL, and the insert size distribution was assessed using an Agilent 2100 Bioanalyzer. Once the insert size met the expectations, the effective concentration of the library was accurately quantified using quantitative PCR to ensure quality. After quality control, libraries were pooled based on effective concentration and target sequencing requirements. The pooled libraries were sequenced on an Illumina platform with PE150 strategy. The sequencing data were analyzed using MetaGeneMark and DIAMOND software. The microbial α‐diversity and β‐diversity were assessed, and the microbial abundance and inter‐group differences were evaluated.

### 2.5 Samples Collection

The dissection of mice was performed in two steps: cardiac perfusion and routine tissue collection. Following anesthesia, the heart was perfused with physiological saline and subsequently with 4% paraformaldehyde. The brain and various intestinal tissues were carefully dissected and fixed in 4% paraformaldehyde. For routine tissue collection, mice were anesthetized, and blood was collected from the heart. Following euthanasia, the brain and intestinal segments were rapidly removed on ice, snap‐frozen in liquid nitrogen, and stored at −80°C. The blood samples were allowed to clot at room temperature for 30 min, followed by centrifugation at 2500 rpm for 10 min at 4°C. Then, the resulting supernatant was transferred into cryogenic tubes and stored at −80°C for further analyses.

### 2.6 Immunostaining Analyses

The brain and intestinal tissues were fixed, dehydrated, embedded in paraffin, and sectioned into slices of 4 μm thickness. Brain sections were subjected to immunohistochemistry (IHC) and immunofluorescence (IF) staining, while intestinal sections were subjected to hematoxylin and eosin (HE) staining and IHC. For IHC, the slides were washed with phosphate buffer solution (PBS) and incubated with 3% hydrogen peroxide at room temperature in the dark for 25 min to inhibit endogenous peroxidase activity. They were then blocked with 3% bovine serum albumin (BSA) for 30 min and incubated overnight at 4°C with primary antibodies, including Aβ, ZO‐1, and occludin. After incubation, the sections were treated with corresponding secondary antibodies for 50 min at room temperature, followed by development using a DAB chromogen kit. For IF staining of brain sections, the slides were blocked with 3% BSA for 30 min, incubated overnight at 4°C with primary antibodies (GFAP and IBA‐1), and stained with DAPI at room temperature for 10 min. The slides were then treated with a tissue autofluorescence quenching reagent for 5 min, washed under running water, and mounted with an anti‐fade mounting medium. The experimental procedures were adapted from previous studies (Qian et al. 2022). IHC images and HE‐stained sections were captured with a Nikon ECLIPSE E100 microscope, while IF images were obtained with a Nikon ECLIPSE C1 confocal microscope. Semi‐quantitative analysis of Aβ, GFAP, IBA‐1, ZO‐1, and occludin was performed using ImageJ software.

### 2.7 Enzyme‐Linked Immunosorbent Assay

The ELISA kits were employed in accordance with the manufacturer's instructions to measure the concentrations of TNF‐α and IL‐6 in mouse serum and brain tissue homogenates, as well as ZO‐1, occludin, claudin‐4, and sIgA in jejunum samples. Standard curves were established using the concentrations and absorbance values of standard samples, and the concentrations of the target biomarkers were subsequently determined.

### 2.8 Statistical Analysis

The data are expressed as mean ± standard deviation (SD). For comparisons among multiple groups, one‐way analyses of variance (ANOVA) or the Kruskal–Wallis test were applied, followed by post hoc tests (Tukey's or Dunn's multiple comparisons test, respectively). All statistical analyses were conducted using GraphPad Prism 9.0 software. Values of *p* < 0.05 were considered statistically significant.

## 3 Results

### 3.1 Crocin Ameliorates Learning and Memory Deficits in 5xFAD Mice

The experimental flowchart is shown in Figure 1A. We evaluated the effects of Crocin on learning and memory in 5xFAD mice using the MWM test. Initially, the strategies used by the mice to locate the platform were categorized into four types: edge‐oriented, random, goal‐directed, and straight‐line. WT group mice progressively adopted the straight‐line strategy as training sessions increased. In contrast, the AD group mice predominantly exhibited edge‐oriented and random strategies, whereas goal‐directed and straight‐line strategies occurred less frequently. The mice were placed in an identical quadrant of the pool, and their swimming trajectories were monitored within 60 s. As shown in Figure 1B, WT group mice predominantly employed the straight‐line strategy, while AD group mice more frequently exhibited edge‐oriented and random strategies. CRL group mice primarily exhibited a random strategy, yet located the platform more quickly than the AD group. Furthermore, both CRM and CRH groups also displayed random strategies; however, CRH group mice exhibited a combination of random and straight‐line strategies, suggesting a modest improvement in learning performance.

As shown in Figure 1C, during the hidden platform test, WT group mice exhibited a significantly shorter escape latency on the first day than the other groups. With continued training, the escape latency gradually decreased across all groups. On the fifth day, the escape latencies were as follows: WT (19.1 s), AD (30.6 s), CRL (29.7 s), CRM (30.8 s), and CRH (27.4 s). Relative to the AD group, the WT group showed the most significant improvement (*p* < 0.05). Among the treatment groups, the CRH group demonstrated the most favorable outcomes, followed by the CRL and CRM groups. These results suggest that Crocin may reduce the escape latency in 5xFAD mice, although the reduction did not reach statistical significance. We subsequently analyzed the average speed of the mice in each group. The results presented in Figure 1D showed that all groups exhibited increased speeds with additional training sessions. Compared with the AD group, the WT group exhibited a significantly faster speed (*p* < 0.05). Although there were no significant differences in speed across the treatment groups, the CRH group showed the highest performance, followed by CRM and CRL. These results suggest that Crocin may enhance the average speed of 5xFAD mice.

Subsequently, we further analyzed the time spent by mice in the target quadrant on the sixth day. The percentage of time spent in the target quadrant was as follows: WT (15.1%), AD (7.4%), CRL (34.1%), CRM (19.6%), and CRH (35.5%), with the AD group spending the least time in the target quadrant (Figure 1E). Although the WT and CRM groups spent more time in the target quadrant compared to the AD group, these differences were not statistically significant. In contrast, the CRL and CRH groups spent a significantly longer time in the target quadrant than the AD group (*p* < 0.01). Collectively, these findings indicate that Crocin treatment increased the time spent in the target quadrant by 5xFAD mice. During the spatial exploration test, the number of platform crossings was compared across groups. As shown in Figure 1F, the results were as follows: WT (2.5), AD (2.25), CRL (2.75), CRM (2.25), and CRH (3.25). No significant difference was observed in the number of crossings between the AD group and the WT, CRL, or CRM groups; the CRM group's performance was comparable to that of the AD group. The CRH group exhibited the highest number of crossings, followed by the CRL and WT groups. This suggests a trend wherein Crocin treatment was associated with an increased number of platform crossings in 5xFAD mice, particularly at a high dose. In summary, the behavioral results suggest that Crocin ameliorates learning and memory deficits in 5xFAD mice, with the high‐dose (CRH) group demonstrating the most robust improvement.

### 3.2 Crocin Alleviates Key Pathological Features in 5xFAD Mice

**FIGURE 2:**
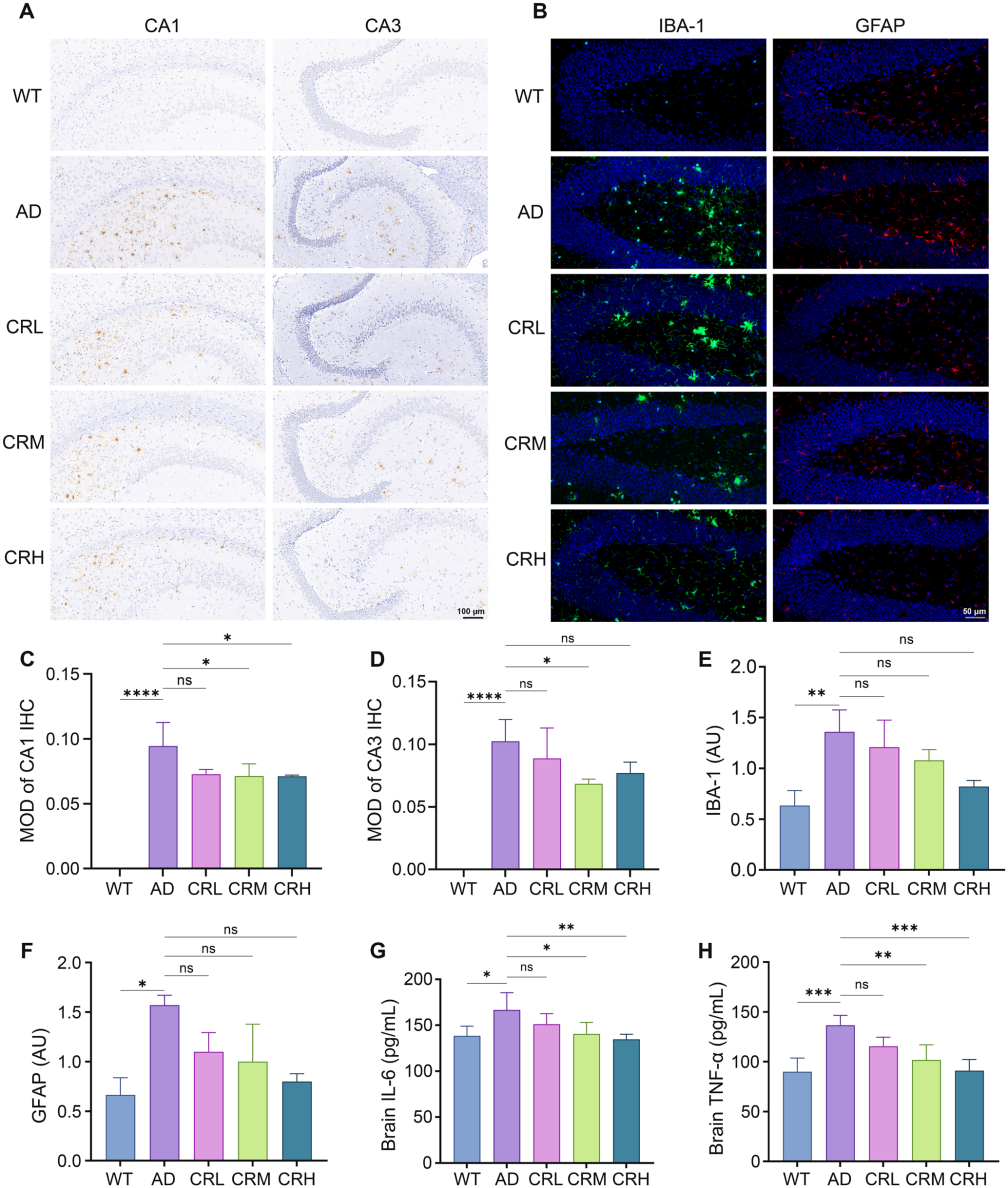
Crocin treatment reduced Aβ pathology and alleviated the proliferation of microglia and astrocytes in 5xFAD mice. (A) Representative photomicrographs of immunostaining of Aβ protein in the hippocampal CA1 and CA3 regions. (B) Immunofluorescence of IBA‐1 and GFAP in the hippocampus. (C, D) Quantification of Aβ protein in the hippocampal CA1 and CA3 regions. (E, F) Quantification of IBA‐1 and GFAP in the hippocampus. (G, H) Expression levels of IL‐6 and TNF‐α in the cerebral cortex. Data are expressed as mean ± SD. We performed statistical analysis with ANOVA followed by Tukey's multiple comparisons test. *p < 0.05, **p < 0.01, ***p < 0.001, ****p < 0.0001; ns, no statistical difference.

The deposition of Aβ plays a central role in the pathogenesis of AD. In the present study, we focused on Aβ pathology as the primary marker due to its early and prominent role in the disease stage under investigation. This peptide comprising 39 to 43 amino acids is proteolytically derived from the amyloid precursor protein (APP) through the sequential cleavage by β‐secretase and γ‐secretase. Aβ can aggregate to form insoluble fibrils, which accumulate as plaques in the brain (Gouras et al. 2015). The 5xFAD transgenic mouse model, which harbors human APP and PSEN1 transgenes with five familial AD mutations, exhibits accelerated Aβ overproduction and deposition (Oakley et al. 2006). We evaluated Aβ deposition in the hippocampal formation of each group of mice using IHC analysis. Representative images revealed no plaques in the CA1 and CA3 areas of hippocampus observed in the WT group mice, whereas plaques were evident in both the AD and Crocin‐treated groups (Figure 2A). To quantify the plaque deposition, CA1 and CA3 subregions of the hippocampus were analyzed using ImageJ software (Figure 2C,D). In the CA1 region, Aβ deposition was highest in the AD group. Crocin treatment induced a non‐significant reducing trend. Similarly, in the CA3 region, the most severe Aβ deposition was observed in the AD group, and Crocin treatment markedly reduced plaque load. Notably, a significant reduction was observed specifically in the CRM group (*p* < 0.05), whereas the CRL and CRH groups showed no significant effects. Collectively, these IHC findings indicate that Crocin treatment mitigates Aβ deposition in the brains of 5xFAD mice, with the most pronounced effect observed in the CRM group.

### 3.3 Crocin Reduces Neuroinflammation in 5xFAD Mice

**FIGURE 3:**
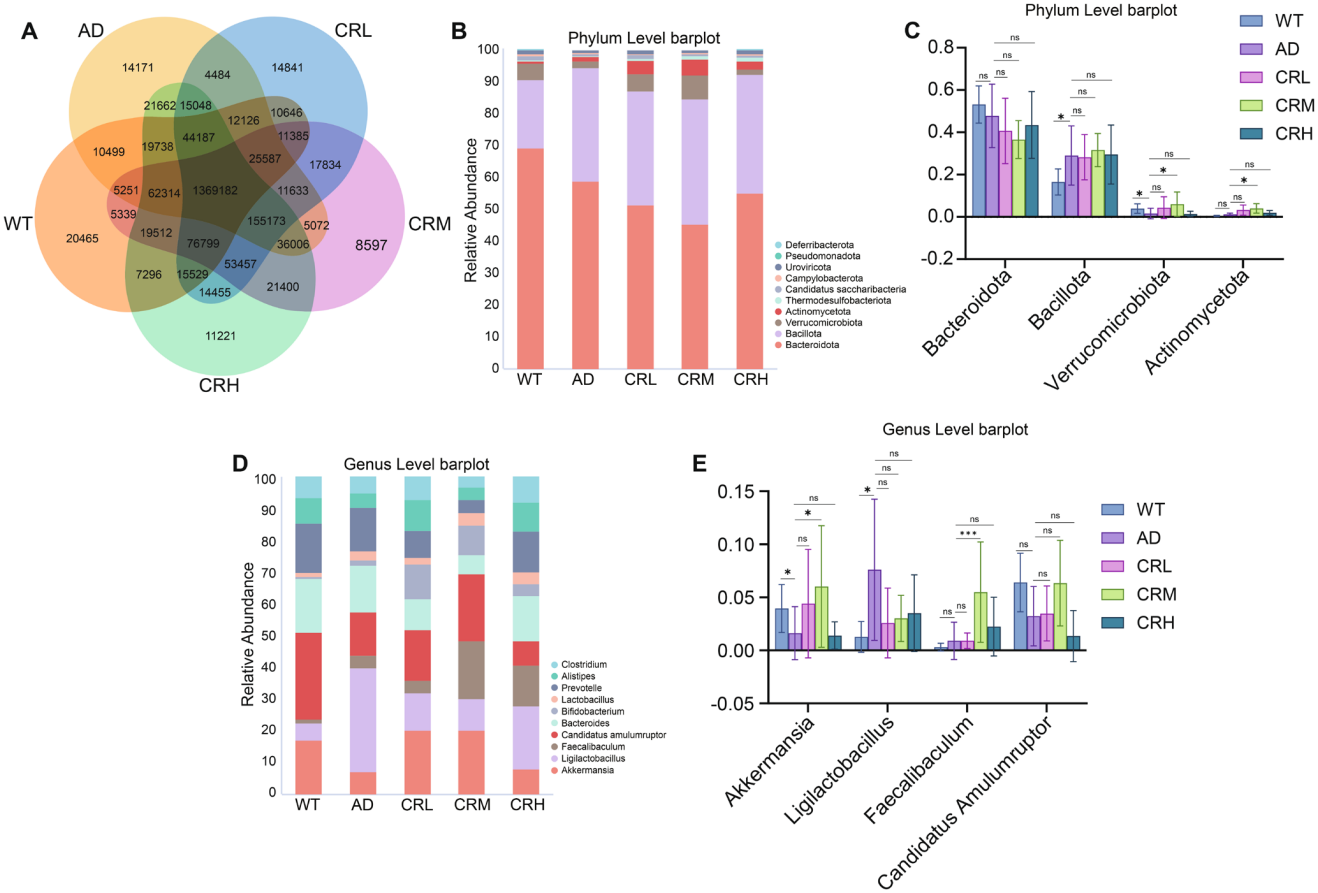
Effects of crocin treatment on the gut microbiota composition of 5xFAD mice. (A) Venn diagrams illustrate the overlap of the characteristic sequence in the gut microbiota among the five groups. (B) The composition of gut microbiota at the phylum level. (C) Four bacterial genera with significant differences at the phylum level. (D) The composition of gut microbiota at the genus level. (E) Four bacterial genera with differences at the genus level. Data are presented as mean ± SD. Statistical analysis was conducted using the Kruskal–Wallis test, followed by Dunn's test for multiple comparisons. *p < 0.05, **p < 0.01, ***p < 0.001, ****p < 0.0001; ns, no statistical difference.

Neuroinflammation is closely linked to the pathogenesis of AD, primarily mediated by microglia and astrocytes (Wu and Eisel 2023). The immune cell markers for microglia and astrocytes are ionized calcium‐binding adaptor molecule 1 (IBA‐1) and glial fibrillary acidic protein (GFAP), respectively (Norden et al. 2016). To evaluate the neuroinflammation, we assessed the activation of microglia and astrocytes by quantifying IBA‐1 and GFAP expression via IF analysis and measured pro‐inflammatory cytokine levels in the brain using ELISA. Representative IF images show IBA‐1 and GFAP expression (Figure 2B). Simultaneously, the fluorescence intensity of IBA‐1 and GFAP was quantified using ImageJ software (Figure 3E,F). IBA‐1 expression was highest in the AD group. In comparison to the AD group, IBA‐1 expression was significantly reduced in the WT group (*p* < 0.01). Crocin treatment reduced IBA‐1 expression, albeit not to a statistically significant extent. Similarly, GFAP expression was highest in the AD group and significantly lower in the WT group (*p* < 0.05). Crocin treatment also reduced GFAP expression; although, similar to IBA‐1, this reduction was not statistically significant. These IF results suggest a potential dose‐dependent trend wherein Crocin may inhibit the activation of microglia and astrocytes.

Microglia and astrocytes activation can stimulate the production of pro‐inflammatory cytokines, including IL‐6 and TNF‐α. To evaluate the effect of Crocin on neuroinflammation, we quantified the levels of IL‐6 and TNF‐α in brain homogenates using ELISA. As shown in Figure 2G, IL‐6 concentrations were highest in the AD group (166.7 pg/mL), followed by the CRL (151.0 pg/mL), CRM (140.5 pg/mL), WT (138.5 pg/mL), and CRH (137.4 pg/mL) groups. Relative to the AD group, IL‐6 levels were significantly lower in the WT group (*p* < 0.05), but not in the CRL group. In contrast, a significant reduction was observed in both the CRM (*p* < 0.05) and CRH (*p* < 0.01) groups. Similarly, TNF‐α levels were elevated in the AD group (136.5 pg/mL) compared to the WT (90.0 pg/mL), CRL (115.5 pg/mL), CRM (101.7 pg/mL), and CRH (91.0 pg/mL) (Figure 2H). Compared to the AD group, TNF‐α levels were significantly lower in the WT (*p* < 0.001), CRH (*p* < 0.001), and CRM (*p* < 0.01) groups. The CRL group did not show a significant difference in TNF‐α levels. Collectively, the IF and ELISA results indicate that Crocin inhibits the activation of microglia and astrocytes, thereby reducing the production of pro‐inflammatory cytokines. Among the treatment groups, the CRM and CRH groups showed the most pronounced effects.

### 3.4 Crocin Changes the Gut Microbiota Composition in 5xFAD Mice

To assess the impact of Crocin on the gut microbiota composition in 5xFAD mice, we first quantified the number of gut microbiota characteristic sequences in the gut. As shown in Figure 3A, the number of characteristic sequences in the fecal samples from the WT, AD, and Crocin‐treated groups was as follows: WT (20,465), AD (14,171), CRL (14,841), CRM (8597), and CRH (11,221). Subsequently, we analyzed the relative abundance of the top 10 bacterial phyla from all fecal samples, as shown in Figure 3B. Among these, *Bacteroidota*, *Bacillota*, *Verrucomicrobiota*, and *Actinomycetota* were the most abundant phyla across all five groups of mice. To assess the effects of Crocin on the phylum level of gut microbiota in 5xFAD mice, we analyzed the relative abundance of these four dominant phyla, as shown in Figure 3C. Compared to the AD group, the relative abundance of *Bacteroidota* and *Verrucomicrobiota* in the WT group was higher, although the differences were not statistically significant. Notably, the relative abundance of *Bacillota* and *Actinomycetota* in the WT group decreased significantly (*p* < 0.05). In the CRL group, the relative abundance of *Verrucomicrobiota* and *Actinomycetota* increased, whereas *Bacteroidota* and *Bacillota* decreased; however, these changes were not statistically significant. In both the CRM and CRH groups, the relative abundance of *Bacillota*, *Verrucomicrobiota*, and *Actinomycetota* increased, whereas *Bacteroidota* decreased. The increase of *Verrucomicrobiota* and *Actinomycetota* in the CRM group was statistically significant (*p* < 0.05), whereas no significant changes were observed in the CRH group. These phylum‐level results suggest that Crocin may enhance the abundance of *Verrucomicrobiota* and *Actinomycetota*, with the most notable effect observed in the CRM group.

At the genus level, the composition of gut microbiota exhibited more pronounced differences. We screened 10 bacterial genera, as depicted in Figure 3D, among which *Akkermansia*, *Ligilactobacillus*, *Faecalibaculum*, and *Candidatus Amulumruptor* were the most abundant genera in the feces of all five groups. To investigate the impact of Crocin on the gut microbiota in 5xFAD mice at the genus level, we analyzed the relative abundance of these four dominant genera, as shown in Figure 3E. Compared with the AD group, the relative abundance of *Akkermansia* significantly increased in the WT group (*p* < 0.05), while the increase in *Candidatus Amulumruptor* was not significant among the five groups. Conversely, *Ligilactobacillus* abundance was significantly reduced (*p* < 0.05), while the decrease in *Faecalibaculum* was not significant. Among the Crocin‐treated groups, only the CRM group showed significant alterations: a marked increase in Akkermansia (*p* < 0.05) and a highly significant increase in Faecalibaculum (*p* < 0.001). Changes in all other genera for the CRL, CRM, and CRH groups were not statistically significant. Collectively, these genus‐level findings indicate that Crocin, particularly at a medium dose (CRM), promotes the abundance of Akkermansia and Faecalibaculum.

### 3.5 Effect of Crocin on Gut Microbiota Diversity

**FIGURE 4:**
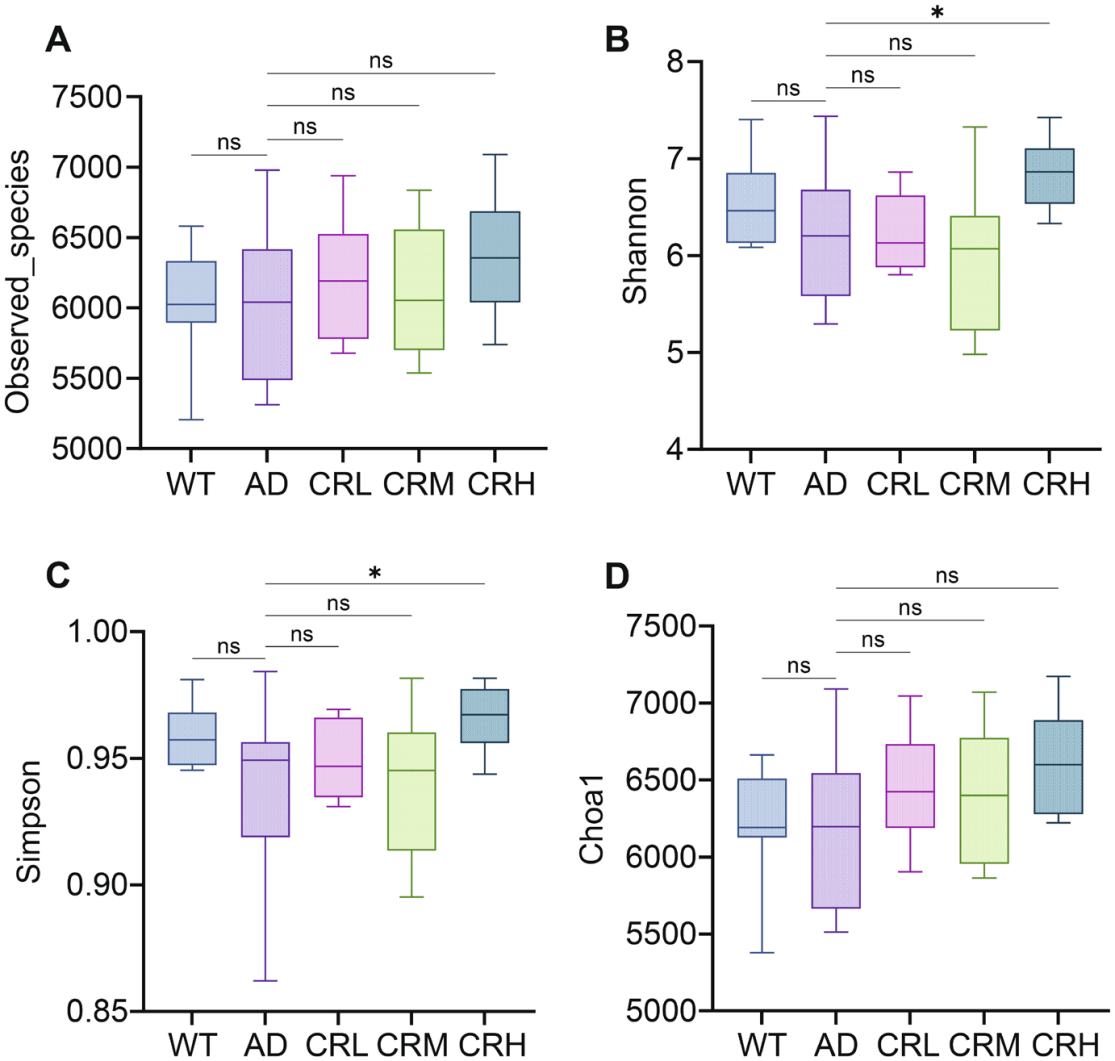
Effects of Crocin treatment on the α diversity of gut microbiota in 5xFAD mice. (A–D) Differences in α diversity (Observed_species index (A), Shannon index (B), Simpson index (C), and Chao1 index (D)) between the five groups. The data are expressed as mean ± SD. Statistical significance was assessed using the Kruskal–Wallis test and followed by Dunn's test for multiple comparisons. *p < 0.05, **p < 0.01, ***p < 0.001, ****p < 0.0001; ns, no statistical difference.

α‐diversity represents the species richness and diversity within individual samples. We evaluated four indices: Observed_species, Chao1, Shannon, and Simpson. The Observed_species index quantifies the number of species detected after randomly sampling a specific amount of sequencing data from the sample. An increased number of observed species suggests a more complex microbial community. As shown in Figure 4A, compared to the AD group, this index decreased in the WT group and increased in the Crocin treatment groups, although no significant differences were observed. The Shannon and Simpson indices estimate microbial diversity within fecal samples. As shown in Figure 4B, compared to the AD group, the Shannon index significantly increased in both the WT and CRH groups (*p* < 0.05) but decreased in the CRL and CRM groups. In Figure 4C, the Simpson index was higher in the WT, CRL, and CRH groups (*p* < 0.05), but decreased in the CRM group compared to the AD group. The Chao1 index estimates microbial richness within fecal samples. As shown in Figure 4D, compared to the AD group, this index decreased in the WT group and increased in the Crocin treatment groups, although no significant differences were observed. The lower Observed_species and Chao1 indices in the WT group compared to the AD group may be attributed to factors such as the age, environment, and tolerance of the mice. Overall, the α‐diversity results suggest that Crocin may enhance gut microbiota diversity in 5xFAD mice, with the CRH group showing the most prominent effect.

**FIGURE 5:**
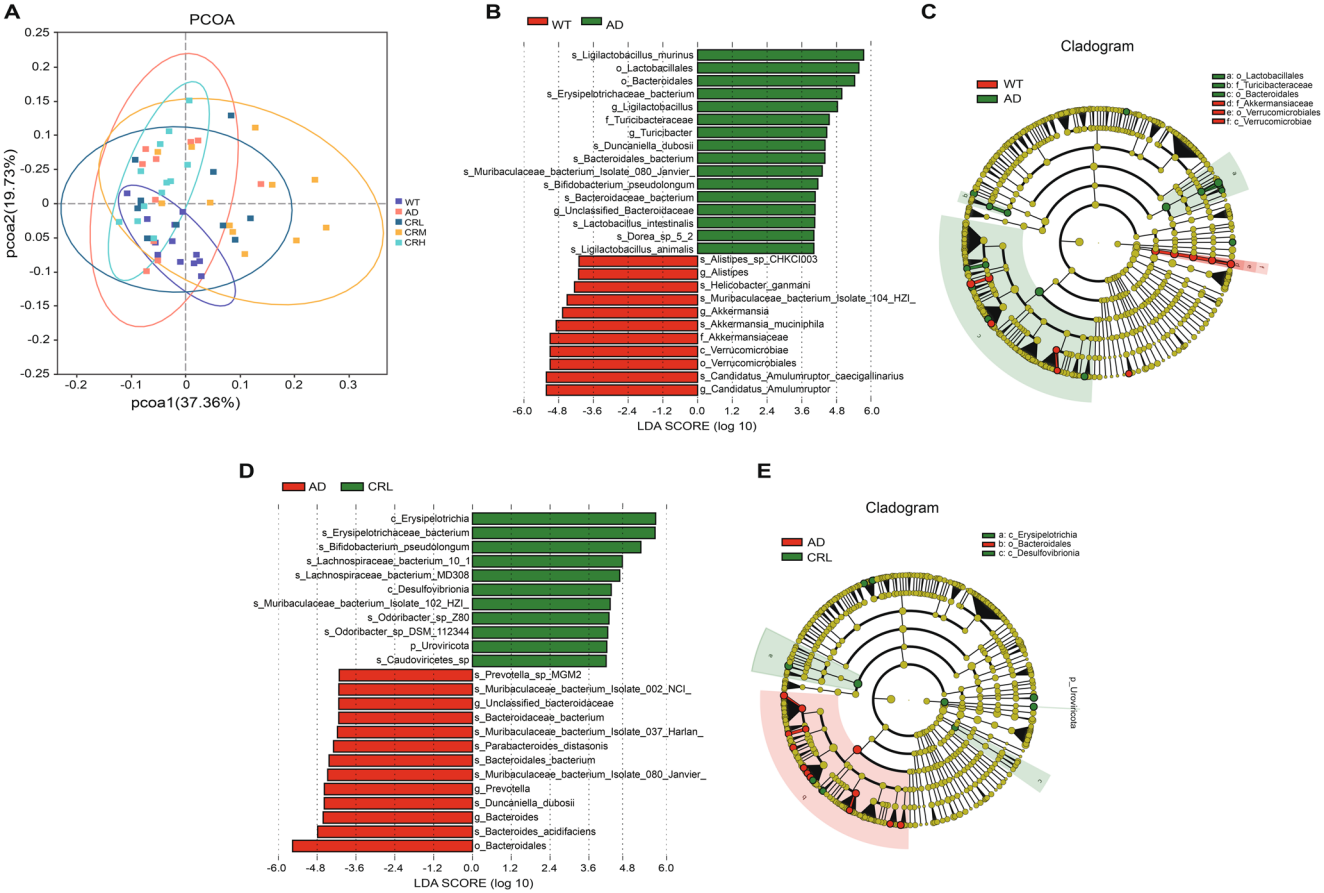
Effects of Crocin treatment on the β diversity and significantly different species among five groups. (A) Principal coordinate analysis (PCoA) for β‐diversity at the genus level, (B, C) linear discriminant analysis (LDA) effect size (LEfSe) analysis revealed significant bacterial differences in gut microbiota between the WT and AD groups, (D, E) the LEfSe identified the taxa with the greatest differences in abundance between the AD and CRL groups. Only taxa with an LDA score greater than 3 are shown.

β‐diversity is employed to evaluate differences in the structure and composition of gut microbiota across diverse samples. Principal coordinates analysis (PCoA) was performed at the genus level based on Bray–Curtis distances, with the two principal coordinate combinations contributing the most (37.36% and 19.73%) selected for plotting. As depicted in Figure 5A, overlap is observed among the groups within the 95% confidence ellipse, suggesting similarity between the samples. However, the WT and CRH samples exhibit a more distinct clustering, implying a higher degree of similarity within these two groups. In contrast, several samples in the CRM group are distinctly separated from the others, signifying variability within this group. The PCoA results demonstrated that Crocin alters the gut microbiota structure in 5xFAD mice, with CRH samples exhibiting a tendency to resemble the WT group more closely.

**FIGURE 6:**
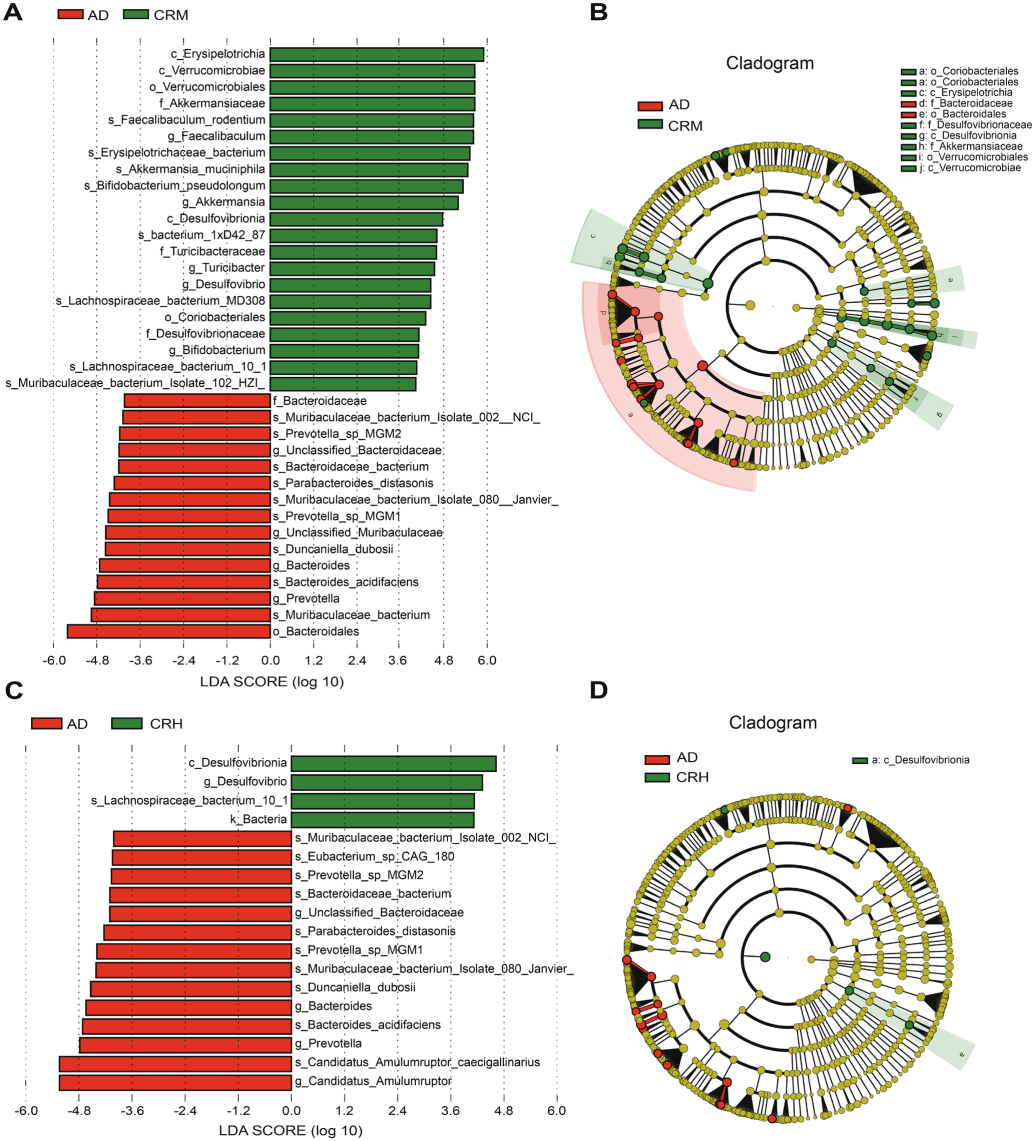
Differential bacterial taxa among the AD, CRM, and CRH groups. (A, B) The LEfSe identified the taxa with the greatest differences in abundance between the AD and CRM groups. (C, D) Significantly different species with an LDA score greater than the preset threshold of 3 between AD and CRH.

To further elucidate the effect of Crocin on gut microbiota diversity, we conducted linear discriminant analysis (LDA) effect size (LefSe) to identify bacteria with significant intergroup differences, using a preset threshold of log_10_ > 3. As shown in Figure 5B, a significant difference in bacterial composition exists between the WT and AD groups. The phylogenetic tree in Figure 5C highlights the key bacteria in the WT group, including *Akkermansiaceae*, *Verrucomicrobiales*, and *Verrucomicrobiae*. In contrast, the AD group exhibits significant bacteria populations, such as *Lactobacillales*, *Bacteroidales*, and *Turicibacteraceae*. Figure 5D illustrates the significant bacterial differences between the AD and CRL groups. The phylogenetic tree in Figure 5E reveals that *Bacteroidales* is a key bacterium in the AD group, whereas important bacteria in the CRL group include *Erysipelotrichia* and *Desulfovibrionia*. Figure 6A illustrates substantial differences in bacterial composition between the AD and CRM groups. Figure 6B identifies key bacterial taxa in the AD and CRM groups, with *Bacteroidaceae* and *Bacteroidales* predominating in the AD group, while *Coriobacteriiales*, *Turicibacteraceae*, *Erysipelotrichia*, *Desulfovibrionaceae*, *Desulfovibrionia*, *Akkermansiaceae*, *Verrucomicrobiales*, and *Verrucomicrobiae* are prominent in the CRM group. Figure 6C presents bacterial differences between the AD and CRH groups, while Figure 6D highlights the absence of significant bacteria in the AD group and the prominence of *Desulfovibrionia* in the CRH group. The LefSe results revealed that Crocin treatment notably enhanced intergroup microbiota differences, with the CRM group exhibiting microbiota changes more closely resembling those of the WT group.

### 3.6 Crocin Ameliorates Intestinal Barrier Injury

**FIGURE 7:**
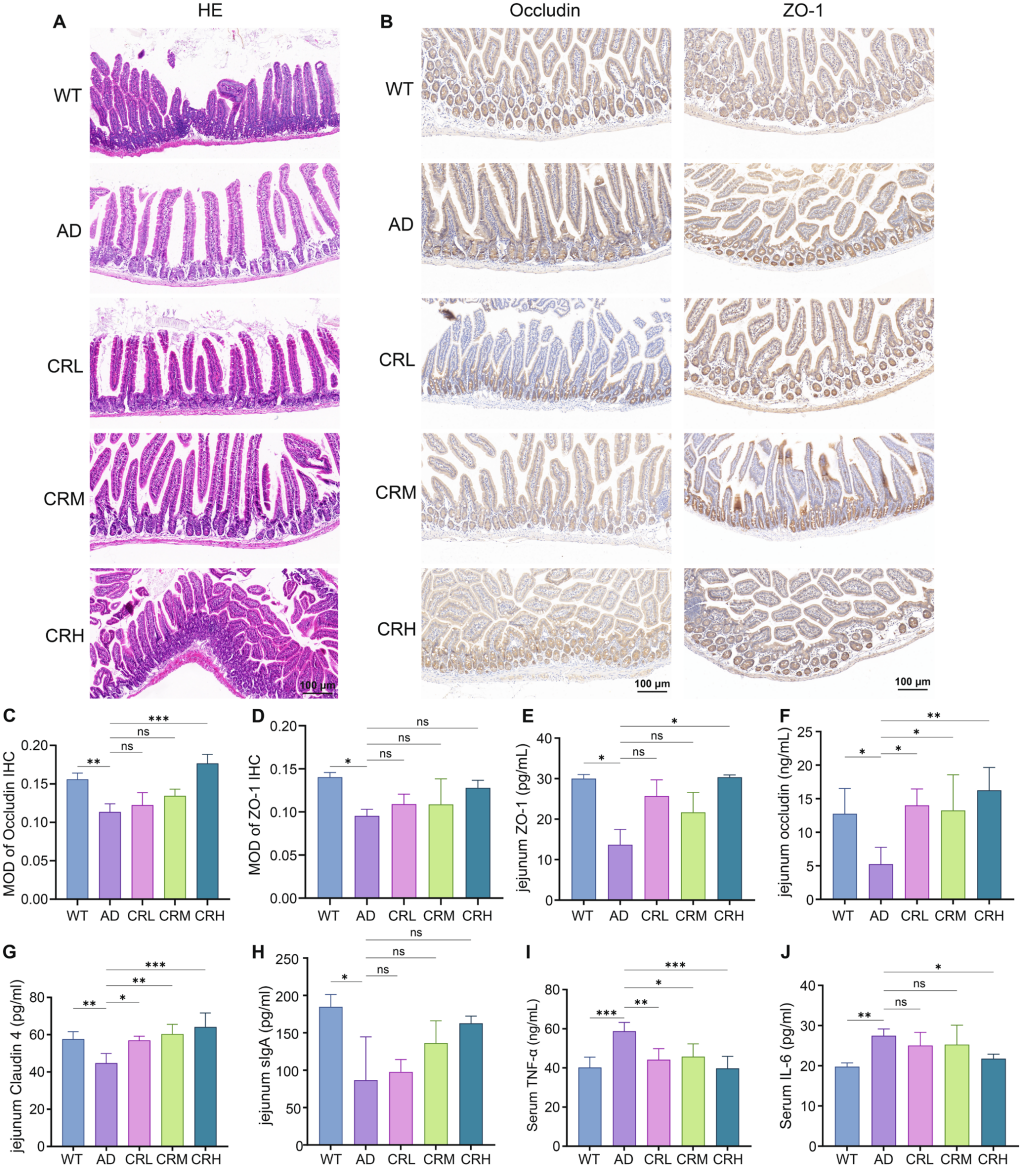
Effects of crocin treatment on intestinal tight junction proteins and inflammation in 5xFAD mice. (A) Representative photomicrographs of HE staining of the jejunum, (B) representative photomicrographs of immunostaining of intestinal tight junction markers occludin and ZO‐1 in the jejunum, (C, D) quantitative image analysis of occludin and ZO‐1 protein expressions in the jejunum was conducted using ImageJ software, (E–H) concentration of ZO‐1, occludin, claudin4, and sIgA proteins in the jejunum, (I, J) serum expression levels of TNF‐α and IL‐6. Data are expressed as the mean ± SD. Data were analyzed using ANOVA and followed by Tukey's multiple comparisons test. *p < 0.05, **p < 0.01, ***p < 0.001, ****p < 0.0001; ns, no statistical difference.

Intestinal mucosal permeability is a critical determinant of intestinal barrier integrity. When compromised, gut‐derived pathogens and endotoxins can translocate across the intestinal mucosa into peripheral circulation, potentially affecting the central nervous system via the gut‐brain axis. To evaluate the effect of Crocin on intestinal barrier function in 5xFAD mice, we examined changes in the jejunal mucosal structure and measured the levels of circulating serum cytokines. Histological analysis revealed a more intact cellular structure in the WT group, whereas the AD group exhibited irregular cell arrangement, enlarged villi, deeper crypts, and a thinner basement membrane. Compared to the AD group, Crocin‐treated mice exhibited morphological improvements, including narrower villi, shallower crypts, and thickened basement membranes; these improvements were most pronounced in the CRH group (Figure 7A). These findings suggest that Crocin mitigates intestinal mucosal damage. The expression levels of the pro‐inflammatory cytokine TNF‐α are shown in Figure 7I. TNF‐α expression was significantly lower in both the WT and all Crocin‐treated groups compared to the AD group (WT: *p* < 0.001, CRL: *p* < 0.01, CRM: *p* < 0.05, CRH: *p* < 0.001). Similarly, expression levels of IL‐6 were also assessed (Figure 7J). The WT group showed significantly lower IL‐6 expression than the AD group (*p* < 0.01). Crocin treatment reduced IL‐6 levels in a dose‐dependent manner, with a significant reduction observed specifically in the CRH group (*p* < 0.05) compared to the AD group. Collectively, these results indicate that Crocin suppresses the expression of peripheral pro‐inflammatory cytokines and helps to protect intestinal barrier function.

We next examined the expression of key intestinal tight junction proteins, which are crucial for maintaining intestinal barrier integrity. Representative IHC staining for the tight junction proteins occludin and ZO‐1 is shown in Figure 7B, alongside quantitative analysis performed using ImageJ software. Quantitative analysis of occludin revealed its lowest expression level in the AD group. Compared to the AD group, occludin expression was significantly elevated in the WT (*p* < 0.01) and CRH (*p* < 0.001) groups; however, the CRL and CRM groups showed no significant increase (Figure 7C). Similarly, quantification of ZO‐1 showed minimal expression in the AD group. Expression of ZO‐1 was significantly increased in the WT group (*p* < 0.05) compared to the AD group, whereas the Crocin‐treated groups exhibited no significant changes (Figure 7D). To further elucidate the effects of Crocin on tight junction proteins, we quantified the expression levels of occludin, ZO‐1, claudin‐4, and sIgA. As shown in Figure 7E, ZO‐1 expression was lowest in the AD group. No significant differences were observed in the CRL and CRM groups compared to the AD group; however, ZO‐1 expression was significantly increased in the WT and CRH groups (*p* < 0.05). Compared to the AD group, occludin levels were significantly elevated in the WT group (*p* < 0.05) and all Crocin‐treated groups (CRL and CRM: *p* < 0.05; CRH: *p* < 0.01) (Figure 7F). Claudin‐4 expression followed a similar pattern, with the lowest level also found in the AD group (Figure 7G). Relative to the AD group, claudin‐4 levels were significantly higher in the WT group (*p* < 0.01) and all Crocin‐treated groups (CRL: *p* < 0.05; CRM: *p* < 0.01; CRH: *p* < 0.001). We also quantified secretory sIgA, a key immunoglobulin in the intestinal mucosa that acts as a first line of defense for barrier function (León and Francino 2022). sIgA levels were lowest in the AD group but were significantly elevated in the WT group (*p* < 0.05). Although Crocin treatment appeared to increase sIgA levels, these changes were not statistically significant (Figure 7H). Collectively, these results suggest that Crocin may enhance intestinal barrier integrity, as evidenced by the upregulation of tight junction proteins, with the most pronounced effects observed in the CRL and CRH groups.

## 4 Discussion

Existing pharmacological treatments for treating AD are frequently costly and accompanied by notable adverse effects. For example, donepezil is associated with side effects such as nausea, vomiting, diarrhea, and bradycardia (Birks and Harvey 2018). Memantine may cause dizziness, confusion, headache, and constipation (McShane et al. 2019). Moreover, more recently approved monoclonal antibodies such as aducanumab and lecanemab have been linked to amyloid‐related imaging abnormalities, including edema and microhemorrhages (Söderberg et al. 2023). Simultaneously, the prices of these two drugs are also quite high (Sinha and Barocas 2022; Nguyen et al. 2024). Hence, there is an urgent need to seek safer and more effective alternative treatment options. Lately, there has been an increasing focus on identifying anti‐AD compounds derived from natural resources. One particularly promising research avenue involves investigating the gut microbiota, gastrointestinal system, enteric nervous system, and central nervous system as an integrated network, which has led to the conceptualization of the “microbiota‐gut‐brain axis” (Lee et al. 2023). This emerging perspective suggests that the pathogenesis of neurological diseases, including AD, may be closely associated with alterations in the gut microbiota. Numerous recent studies emphasize the bidirectional regulatory relationship between the gut microbiota and the central nervous system. On one hand, the central nervous system regulates gut motility and secretion, thereby influencing the composition and ecology of the gut microbiota. On the other hand, the gut microbiota, through its metabolites, can impact brain development and function (Socała et al. 2021). Consequently, the study of neurological diseases has expanded beyond a sole focus on the brain to encompass alterations in the gut microbiota and its potential role in disease pathogenesis. Indeed, recent investigations have revealed a significant correlation between changes in the gut microbiota and the development of AD in patients (Ling et al. 2021).

TCM has been employed for millennia in China and remains a valuable source of natural medicinal resources, characterized by both significant biological activity and safety. Among various TCM compounds, saffron contains crocin, one of its primary components (Abu‐Izneid et al. 2022). While saffron is primarily regulated as a medicinal material in China, its application as a food ingredient enjoys a long history and widespread acceptance in the global food industry. Saffron is universally recognized as a safe and valuable spice. For instance, it holds a well‐established position in the culinary traditions of many European, Middle Eastern, and Asian countries. Notably, the European Food Safety Authority (EFSA) and the U.S. Food and Drug Administration (FDA) classify saffron as generally recognized as safe (GRAS) for use as a spice, seasoning, or natural flavoring (Abu‐Izneid et al. 2022). This international precedent firmly supports the concept of utilizing saffron and its constituents like crocin beyond purely medicinal contexts, aligning it with the global trend of developing functional foods. Furthermore, toxicological studies have indicated a favorable safety profile for crocin and saffron extracts. For example, studies report that the median lethal dose of saffron is relatively high, and no significant adverse effects are observed at doses well above those used for nutritional purposes (Khazdair et al. 2015). Finally, regarding feasible dietary intake, clinical trials have investigated the efficacy and safety of saffron extracts in humans (Ayati et al. 2020). To systematically assess the mechanism of crocin in AD, we established a 5xFAD mouse model and investigated crocin's effects through behavioral experiments, histological analysis, and microbiome profiling. Our results demonstrated that crocin significantly alleviated cognitive impairments in 5xFAD mice, with the CRH group exhibiting superior outcomes compared to the CRL and CRM groups. Previous studies have indicated that crocin possesses neuroprotective effects and may improve cognitive dysfunction (Baghishani et al. 2018). Furthermore, no significant adverse reactions were observed, underscoring the safety of crocin, which is a key rationale for conducting this study.

In this study, Crocin was demonstrated to enhance the cognitive function of 5xFAD mice, reduce hippocampal Aβ deposition, and mitigate neuroinflammation. The 5xFAD transgenic mice exhibit elevated levels of Aβ42, a characteristic hallmark of AD (Nehra et al. 2024). Compared to saline treatment, Crocin administration enhanced the cognitive performance of the 5xFAD mice and markedly reduced Aβ42 deposition in the hippocampus, particularly in the CA1 and CA3 regions, thereby confirming its therapeutic potential. Neuroinflammation, typically marked by the proliferation of microglia and astrocytes, constitutes a critical aspect of AD pathology. IBA‐1 and GFAP are widely recognized markers for microglia and astrocytes, respectively, and the activation of these glial cells triggers the upregulation of pro‐inflammatory cytokines (Norden et al. 2016; Lanfranco et al. 2021). In patients with AD, studies have demonstrated that microglial markers including IBA‐1, Transmembrane protein 119 (TMEM119), and P2ry12 are altered, characterized by reduced TMEM119‐positive microglia, loss of P2ry12 expression, and increased IBA‐1 positivity, collectively indicating microglial activation (Kenkhuis et al. 2022). Moreover, GFAP overexpression has been observed in the brains of both AD patients and AD mouse models, suggesting that astrocyte proliferation (Roveta et al. 2024). In our study, IF analysis of IBA‐1 and GFAP expression revealed that the AD group exhibited the highest levels in the hippocampus, whereas the WT group displayed minimal expression. Crocin treatment resulted in a marked reduction in the expression of both markers across all experimental groups, with the CRH group demonstrating the most pronounced effect. These IF findings suggest that Crocin inhibits the proliferation of microglia and astrocytes in a dose‐dependent manner.

An expanding body of evidence increasingly supports the crucial role of the gut microbiota in the pathogenesis of AD. Due to its high molecular weight, Crocin must undergo metabolic conversion into crocic acid to be absorbed into the bloodstream. Therefore, the therapeutic effects of Crocin are likely mediated through its impact on the gut microbiota, a hypothesis that is strongly supported by the results of this study. Following Crocin treatment, a significant increase in α‐diversity was observed, and β‐diversity analysis revealed that Crocin treatment shifted the gut microbiota composition in 5xFAD mice towards that of the WT group. These findings suggest that Crocin exerts a regulatory effect on the structure of the gut microbiota. Furthermore, Crocin treatment notably increased the abundance of *Verrucomicrobiota* and *Actinomycetota* at the phylum level, as well as *Akkermansia* and *Faecalibaculum* at the genus level. *Akkermansia* and *Faecalibaculum* are especially noteworthy. *Akkermansia*, a member of the *Verrucomicrobiota* phylum, is known to downregulate pro‐inflammatory cytokines and inhibit inflammation in mouse models of dextran sulfate sodium‐induced chronic colitis (Bian et al. 2019). Furthermore, *Akkermansia* has been shown to regulate the gut microbiota and suppress neuroinflammation. Additionally, *Akkermansia* and *Firmicutes* are widely recognized as pivotal producers of SCFAs, such as butyrate, propionate, and acetate (Martín et al. 2023). SCFAs are crucial metabolites that mediate the communication along the gut–brain axis. Butyrate, in particular, serves as a primary energy source for colonocytes, strengthens the gut barrier, and exerts potent anti‐inflammatory effects both locally and systemically (Silva et al. 2020; Chen et al. 2024). Furthermore, propionate and acetate can cross the blood–brain barrier, where they influence microglial maturation, neuroinflammation, and overall brain health (Fock and Parnova 2023). Although we were unable to directly measure SCFA levels in this study due to sample limitations, the observed microbial shifts strongly suggest a concomitant increase in SCFA production as a plausible mechanistic pathway for the neuroprotective effects witnessed in our animal models. This proposed mechanism warrants explicit investigation in future studies through direct quantification of SCFAs in serum, feces, and brain tissue.

Notably, when strains of *Faecalibaculum* isolated from healthy human donors were administered to AD model mice, cognitive impairments were mitigated. The abundance of *Faecalibaculum* is reduced in AD patients relative to healthy individuals, consistent with our findings. Beyond their roles in modulating the gut microbiota and influencing neurological disorders, *Akkermansia* and *Faecalibaculum* also enhance intestinal barrier function (Martín et al. 2015; Liu, Yue, et al. 2020). Given their involvement in gut microbiota modulation and intestinal barrier enhancement, we evaluated intestinal barrier markers in 5xFAD mice to determine the impact of Crocin treatment on barrier integrity. Crocin treatment increased the thickness of the intestinal basement membrane, reduced villus spacing, decreased crypt depth, and upregulated the expression of tight junction proteins, including ZO‐1, occludin, and claudin‐4. Given the significant increase in the abundance of *Faecalibaculum* and *Akkermansia*, along with the inhibitory effects of *Akkermansia* on neuroinflammation, we hypothesize that Crocin improves cognitive dysfunction and mitigates pathological features in 5xFAD mice by modulating the gut microbiota, enhancing intestinal barrier function, and suppressing neuroinflammation. Beyond these correlative observations, a critical question remains regarding how Crocin directly influences the gut microbial composition. While our study was not designed to definitively pinpoint the exact molecular targets, we can speculate on several potential mechanisms based on existing literature. First, Crocin and its metabolites may interact with bile acid signaling pathways. Structurally, Crocin shares similarities with bile acids and may act as a ligand or modulator for the Farnesoid X Receptor (FXR), a nuclear receptor highly expressed in the intestine and liver (Chiang and Ferrell 2022). FXR activation plays a central role in regulating bile acid homeostasis, which in turn exerts selective antimicrobial effects and can shape the gut microbiota, potentially favoring the growth of specific beneficial bacteria. Second, Crocin might possess selective antimicrobial or prebiotic properties. Certain bioactive compounds can inhibit the growth of pathobionts while promoting the proliferation of health‐associated bacteria like *Akkermansia*, without necessarily being metabolized themselves (Li et al. 2025). Finally, the effects may be indirect via the host. Crocin's well‐documented anti‐inflammatory and antioxidant properties could ameliorate gut barrier dysfunction and reduce mucosal inflammation, thereby creating a favorable environment for a beneficial microbial ecosystem to thrive (Bastani et al. 2022). Future mechanistic studies, including in vitro cultures with Crocin, fecal microbiota transplantation experiments, and targeted metabolomics, are essential to validate these hypotheses and identify the primary targets of Crocin in microbiota regulation.

To further contextualize our findings on Crocin, it is instructive to compare its efficacy and mechanisms with other well‐characterized natural compounds also investigated for AD therapy, such as gastrodin and curcumin. Gastrodin, the primary bioactive component of Gastrodia elata, has demonstrated neuroprotective effects primarily through its anti‐oxidative and anti‐inflammatory activities, alongside modulation of neurotransmitters like GABA and dopamine (Fasina et al. 2022). However, while its direct neuroeffects are established, evidence for a primary role in gut microbiota modulation remains less explored compared to its central actions. Curcumin, from turmeric ( *Curcuma longa* ), shares crocin's strong anti‐inflammatory and anti‐oxidant properties. It has been more extensively studied in the context of the gut‐brain axis, with research indicating its ability to ameliorate gut dysbiosis, reduce intestinal permeability, and improve cognitive function in animal models of AD (Lou et al. 2024). Its mechanisms often involve the suppression of NF‐κB and TLR4 signaling pathways. However, its clinical translation is often hampered by exceedingly low oral bioavailability. In contrast, our study demonstrates that crocin's beneficial effects are significantly associated with the restoration of specific microbial taxa, particularly an increase in *Akkermansia* and *Faecalibaculum*. This shift was correlated with the amelioration of reduced TNF‐α, IL‐6, Aβ plaque deposition, and memory deficits. This suggests that although these compounds may share overarching anti‐AD properties, the precise pathways through which they modulate the gut‐brain axis and confer neuroprotection are compound‐specific. The novel contribution of our work lies in delineating this unique gut microbiota‐mediated mechanism for crocin, distinguishing it from other herbal analogs and highlighting its potential as a multi‐target therapeutic agent for AD.

Although the findings of this study are promising, several limitations should be acknowledged. Firstly, the 5xFAD transgenic mouse model, which primarily replicates the amyloid pathology observed in familial AD, may not fully capture the complexity of sporadic AD in humans. Future research using alternative models, such as tauopathy models or those incorporating both amyloid and tau pathologies, will offer a more thorough evaluation of Crocin's therapeutic potential. Secondly, while our data suggest a potential gut‐brain axis mechanism, the exact causal relationships and the specific microbial metabolites involved have yet to be determined. To establish causality and identify the critical effector molecules, further studies, including fecal microbiota transplantation from Crocin‐treated mice to untreated AD mice, along with metabolomic analyses, are needed. Finally, the clinical relevance of our findings to human patients requires additional validation through well‐designed clinical trials to determine the optimal dosage, long‐term safety, and efficacy of Crocin.

## 5 Conclusion

This study demonstrated that Crocin treatment significantly alleviated cognitive deficits and neuropathology in a 5xFAD mouse model of AD. Crucially, we established that these neuroprotective effects were accompanied by a profound modulation of the gut microbiota and strengthening of the intestinal barrier. These findings provided compelling in vivo evidence that underscores the pivotal role of the gut‐brain axis in AD pathogenesis. Simultaneously, we suggested that targeting the gut microbiota could be a viable therapeutic strategy, moving beyond traditional central nervous system‐centric approaches. Crocin, a natural compound with a known safety profile, presents itself as a promising novel therapeutic or adjunctive agent. A promising translational approach involves developing synergistic formulations combining crocin with probiotic strains such as *Akkermansia muciniphila* . Our work paved the way for further clinical investigations into microbiota‐targeting interventions for neurodegenerative diseases. In summary, our data not only illustrated Crocin's potential as a therapeutic agent but also significantly advanced our understanding of AD as a systemic disorder intimately linked to gut health.

## Author Contributions

**Zhiyan Zou:** conceptualization (equal), data curation (equal), software (equal), supervision (equal), writing – original draft (equal). **Dan Lei:** data curation (equal), software (equal), validation (equal), visualization (equal). **Xilian Wang:** investigation (supporting), validation (supporting). **Yuan Yin:** data curation (supporting), formal analysis (supporting), supervision (supporting), writing – review and editing (supporting). **Xin Di:** investigation (supporting), methodology (supporting), writing – review and editing (supporting). **Hong Li:** funding acquisition (supporting), investigation (supporting). **Xiaoan Li:** conceptualization (equal), funding acquisition (equal), project administration (equal).

## Ethics Statement

The study adhered to all applicable international, national, and institutional guidelines for animal care and use. All experimental protocols were approved by the Animal Ethics Committee of the Mianyang Central Hospital with permission number S20240205‐01 and were conducted in accordance with the ethical standards outlined in the Declaration of Helsinki.

## Consent

The authors have nothing to report.

## Conflicts of Interest

The authors declare no conflicts of interest.

## Data Availability

The data presented in this study is available on request from the corresponding author.
